# Effects of Dry and Wet Ultrafine Grinding on the Extraction Efficiency and Biological Activities of Polyphenols from Peach Pomace

**DOI:** 10.3390/foods15152682

**Published:** 2026-07-30

**Authors:** Bohua Zhang, Qi Fan, Chao Ma, Chongdui Wang, Maoyu Wu, Fuxia Hu, Li Wang, Ziyu Ren, Mayire Abulikemu, Ming Zhang

**Affiliations:** Jinan Fruit Research Institute of All China Federation of Supply and Marketing Cooperatives, Jinan 250014, China; zbh19901127@163.com (B.Z.); 15169071876@163.com (C.W.);

**Keywords:** peach, ultrafine grinding, polyphenols, antioxidant, anti-inflammatory

## Abstract

In order to screen the ultrafine grinding methods which are conducive to polyphenol release and biological activity, the peach pomace was respectively treated by dry ultrafine grinding and wet ultrafine grinding, and then the polyphenol extraction, component identification and functional activity evaluation were carried out. The results showed that the content and purity of polyphenols increased by 23.3% and 25.6% respectively after wet ultrafine grinding compared with coarse grinding. The results of HPLC-MS identification showed that chlorogenic acid, D-(-)-quinic acid and catechins were the main polyphenols in the dry superfine grinding residue. However, benzoic acid, quercetin-3-β-D-glucoside and rutin were the main components in the wet superfine grinding residue. The results of antioxidant and anti-inflammatory activity evaluation showed that the peach pomace extract obtained by wet ultrafine grinding had better antioxidant activity (DPPH· and ABTS^+^· scavenging rate and total reducing power reached 80.16%, 47.07% and 1.112 respectively) and anti-inflammatory activity. In the study of *α*-glucosidase inhibitory activity, it was found that the polyphenols prepared by wet ultrafine grinding had higher inhibitory effect on *α*-glucosidase (80.22%), and based on the enzyme inhibition kinetics, it showed reversible competitive inhibition on *α*-glucosidase. Finally, in terms of antibacterial activity, the polyphenol extract prepared by wet ultrafine grinding had the strongest inhibitory effect on Staphylococcus aureus, Escherichia coli and Bacillus subtilis, and its minimum inhibitory concentrations were 3.13, 3.13 and 12.50 mg/mL, respectively. In conclusion, wet ultrafine grinding is more conducive to the efficient extraction of active polyphenols from peach pomace and its biological activity.

## 1. Introduction

Peach (*Prunus persica* L.) is a peach plant of rosaceae. It is a characteristic fruit of Shandong Province, China. It is known as “China longevity peach” and “Xiantao”. It is famous for its good quality, such as upright fruit shape, large single fruit, tender meat, juicy and strong taste [1]. At present, the peach processing industry is still dominated by primary processed products such as cans, candied fruit, fruit pulp and fruit juice, and a large number of by-products are produced in the processing process [2]. This part of the by-products is rich in polyphenols, polysaccharides, monounsaturated fatty acids, peptides, lecithin, vitamin E and other bioactive components, and has significant nutritional and functional value [3,4]. However, at present, these resources have not been effectively recycled and utilized, which not only causes waste of valuable raw materials, but also restricts the extension of the peach industry chain and the improvement of the overall added value [5,6]. Therefore, strengthening the research on the key technologies of peach deep processing and the comprehensive utilization of by-products, developing high value-added products and realizing the efficient utilization of the whole fruit have become the key scientific problems and industrial bottlenecks to promote the sustainable development of peach industry and enhance market competitiveness.

In the deep processing of fruit and the resource utilization of by-products, the traditional preparation technology mainly involves solvent extraction [7], hot reflux [8], pressing and conventional drying and milling methods, which has the characteristics of simple operation, low equipment investment and wide applicability, and is widely used in fruit processing. However, most of the above technologies rely on long-term heat treatment or organic solvent assistance, which can easily lead to degradation, oxidation and solvent residue of thermal-sensitive components, low extraction efficiency and high energy and water consumption [9]. Conventional mechanical crushing is difficult to effectively destroy the dense structure of plant cell wall, which hinders the dissolution of intracellular functional components such as bound polyphenols, insoluble dietary fiber and fat soluble nutrients, and the comprehensive utilization rate of resources is low [10]. In addition, the traditional process is prone to quality deterioration and affects the stability of the product when dealing with the peach pomace rich in heat-sensitive substances. The above limitations seriously restrict the high-value transformation of by-products, which is difficult to meet the development requirements of modern food industry for energy efficiency and green manufacturing. Therefore, it is urgent to explore new processing technologies to realize the efficient release and accurate utilization of functional components of peach pomace.

Ultrafine grinding technology is a new processing technology in recent years, which is usually used in food processing, biotechnology, pharmaceutical industry and other fields [11,12]. Compared with the traditional preparation technology, the ultrafine grinding technology has greater potential and development space. As a new pretreatment method, ultrafine pulverization technology is commonly used in low-temperature wall-breaking ultrafine pulverizers, wet ultrafine pulverizers and other equipment for ultrafine pulverization, which refers to the process of crushing material particles above 3 mm to less than 10–25 μm. Its principle is to generate strong shear force and impact force to break the particles, reduce the particle size, increase the specific surface area, and expose more groups, significantly damage the plant cell wall structure, so that the powder has a larger surface area and porosity, and finally make the particles have higher interfacial activity, so as to improve the dissolution efficiency of functional components and increase the content of some nutrients [13,14,15]. At present, dry superfine grinding and wet superfine grinding are the main application forms of this technology. Dry superfine grinding mainly includes air flow type, ball milling type, high-frequency vibration type, impact type, etc. It is a physical processing technology that can crush materials into small particles without adding any liquid medium in the dry state, and realize superfine materials through mechanical force. Dry superfine grinding has the advantages of simple operation, low energy consumption and being suitable for large-scale continuous production. High heat is generated due to friction in the grinding process, and high temperature will lead to the destruction and loss of heat-sensitive components in materials. Wet ultrafine grinding mainly includes colloid mill, high-pressure homogenizer, etc. It is a technology to pulverize materials to the micron or nanometer level in liquid medium, completely destroy the cell wall structure and reduce the dissolution resistance of substances in cells [16]. Through the buffer and dispersion of liquid medium, the thermal effect in the grinding process can be reduced, and the degradation of thermal-sensitive components can be reduced. However, the wet superfine grinding process is relatively complex, with high cost and energy consumption.

*Prunus persica* L.’s large number of by-products from peel residue produced during deep processing are rich in polyphenol bioactive components, which are important potential resources for realizing high-value utilization of whole fruit. As a new cell wall-breaking technology, ultrafine grinding can significantly improve the dissolution rate of functional components [17,18]. However, at present, the research on the efficient extraction and functional evaluation of polyphenols in peach pomace is still relatively limited, and the influence mechanism of different ultrafine grinding technologies on its biological activity is still unclear.

To address these limitations, this study investigates the application of dry and wet ultrafine pulverization technologies on peach pomace to maximize the recovery and bioactivity of polyphenolic compounds. The primary objective is to systematically compare the effects of these two distinct grinding methods on polyphenol yield, purity, and chemical composition. Furthermore, we comprehensively evaluate the functional properties of the resulting extracts by assessing their antioxidant capacities (DPPH·, ABTS^+^·, and total reducing power), anti-inflammatory activities (using a RAW264.7 cell model), α-glucosidase inhibitory kinetics, and antibacterial effects. The novelty of this research lies in elucidating how different mechanical stress environments and temperature-buffering conditions (dry versus wet grinding) dictate the release, preservation of heat-sensitive components, and ultimate biological efficacy of peach pomace polyphenols. By establishing a comprehensive functional evaluation system, this work aims to identify the optimal processing technology for upcycling peach by-products. Ultimately, this provides a critical theoretical foundation and practical strategy for the high-value, sustainable manufacturing of fruit processing waste.

## 2. Materials and Methods

### 2.1. Materials and Chemicals

Fresh peaches (*Prunus persica* L., cultivar: Mengyin) at commercial maturity were purchased directly from a local orchard in Jinan, Shandong Province, China. All fruits were selected from a single harvest batch to minimize biological variance, ensuring uniform size, color, and the absence of mechanical damage or visual defects. The fresh peaches were transported to the laboratory within 2 h of harvesting and temporarily stored at 4 °C. The initial processing (cleaning, enucleating, and crushing) was commenced within 24 h. The initial moisture content of the resulting fresh peach pomace was determined to be approximately 85.5 ± 1.2% (*w*/*w*).

Ascorbic acid, sodium carbonate, folinol, absolute ethanol, sodium hydroxide, hydrochloric acid, potassium ferricyanide, ferric chloride and trichloroacetic acid were purchased from Sinopharm Chemical Reagent Co., Ltd. (Shanghai, China), 1,1-diphenyl-2-picrylhydrazine free radical, 2,2′-Hydrazine bis (3-ethylbenzothiazoline-6-sulfonic acid) diamine salt were purchased from Shanghai McLean Biochemical Technology Co., Ltd. (Shanghai, China), AB-8 macroporous adsorption resin was purchased from Zhengzhou Hecheng New Materials Technology Co., Ltd. (Zhengzhou, China), DMEM high glucose medium and lipopolysaccharide (LPS) were purchased from Sigma-Aldrich (St. Louis, MO, USA), and mouse macrophage line RAW 264.7 was purchased from at Wuhan boster Biotechnology Co., Ltd. (Wuhan, China); the above chemical reagents are analytically pure. The α-glucosidase (EC 3.2.1.20, from *Saccharomyces cerevisiae*) and *p*-nitrophenyl-α-D-glucopyranoside (PNPG) were purchased from Sigma-Aldrich (St. Louis, MO, USA).

### 2.2. Equipment

FD-2 Freeze dryer (Shanghai Bilang Instrument Manufacturing Co., Ltd., Shanghai, China), BW-W200 Low-temperature wall-breaking superfine pulverizer (Shandong Baishun Machinery Co., Ltd., Weifang, China), 1500 Wet superfine pulverizer (UNISOL Trading Shanghai Co., Ltd., Shanghai, China), Ultim3000 High-performance liquid chromatograph (Thermo Fisher Scientific, Waltham, MA, USA), Orbitrap Exploris 480 Ultra-high-resolution mass spectrometer (Thermo Fisher Scientific, USA).

### 2.3. Pretreatment with Different Superfine Grinding Methods

After cleaning, enucleating, cutting, and crushing, the fresh peach was filtered to separate the juice, and the remaining solid matrix (a mixture of peel and fibrous pulp) was collected as the peach pomace. To ensure a uniform baseline for all treatments, this fresh pomace was uniformly dried at 50 °C for 6 h and then crushed by a pulverizer for 1 min to obtain the pre-dried coarse crushed peach pomace, which was used as the control group.

For the dry superfine grinding treatment, a portion of this pre-dried coarse pomace was processed using a low-temperature wall-breaking superfine pulverizer. The equipment operated at a high rotation speed of 25,000 rpm. The grinding chamber was equipped with a continuous cooling water circulation system to strictly maintain the process temperature at 0 °C. The processing time was set to 30 min, yielding a final dried superfine powder with a nominal particle size capable of passing through a 300-mesh sieve (approximately 45 μm).

For the wet pulverization treatment, another portion (400 g) of the identical pre-dried coarse pomace was mixed with deionized water at a solid-to-liquid ratio of 1:3 (*w*/*v*), with the water serving solely as a liquid grinding medium. This suspension was processed through a colloidal mill. The mill was operated at a high shear rotation speed of 2880 rpm with a stator–rotor clearance adjusted to 10 μm. To ensure thorough cell wall disruption, the mixture was circulated at a flow rate of approximately 2.0 L/min for three consecutive cycles. The process temperature was kept below 35 °C. The final homogenous suspension was defined as the wet superfine pulverized peach pomace, which was then concentrated using a rotary evaporator to obtain the final wet pulverized sample for subsequent extractions.

### 2.4. Extraction of Polyphenols from Peach Pomace

To uniformly evaluate the effects of different pulverization pretreatments on polyphenol release, standard extraction conditions were established based on preliminary experiments. Specifically, the peach pomace subjected to coarse grinding and dry ultrafine grinding was dissolved in a 50% ethanol solution at a solid-to-liquid ratio of 1:50, whereas the wet ultrafine grinding sample was dissolved in a 50% ethanol solution at a ratio of 1:10. Subsequently, the mixtures were incubated in a thermostatic shaking water bath at 60 °C for 3 h to facilitate the complete release of polyphenols. Following the extraction phase, the mixtures were centrifuged at 5000 rpm for 15 min at room temperature to precipitate the solid residue. The separated supernatants were collected and then concentrated using a rotary evaporator at 50 °C. After completion, it was transferred to a volumetric flask to obtain peach polyphenol extract, which were coarse grinding of peach polyphenols (CPP), dry ultrafine grinding of peach polyphenols (DPP) and wet ultrafine grinding of peach polyphenols (WPP).

### 2.5. Determination of Polyphenol Content

A total of 0.01 g gallic acid standard was accurately weighed and diluted to 100 mL to obtain 0.1 mg/mL standard stock solution. Then 0, 0.06, 0.12, 0.18, 0.24, 0.3 mL of the solution were accurately absorbed, and water was added to 0.3 mL. Then 1.2 mL of water, 0.5 mL of Folin phenol reagent and 1.0 mL of 7.5% Na_2_CO_3_ solution were added successively. After mixing, the reaction was avoided for 1 h. The absorbance was measured at 770 nm, and the standard curve was drawn. Finally, 0.3 mL of polyphenol extract diluted four times was absorbed, and the absorbance was determined according to the same method, in triplicate. The total polyphenol content was calculated according to the standard curve and expressed as milligrams of gallic acid equivalents per gram of dry peach pomace (mg GAE/g DW) [19].

### 2.6. Purification and Purity Determination of Polyphenols

Based on the method of Odeh et al. (2017) [20] with appropriate modifications, the polyphenols from peach pomace were purified by AB-8 macroporous resin. It was placed in anhydrous ethanol at room temperature, fully soaked for 24 h, and then washed with deionized water until tasteless and no white turbidity. Then it was soaked in 5% HCl solution for 6 h and washed to neutral with deionized water. Finally, after soaking in 5% NaOH solution for 6 h, use deionized water to wash to neutral, and use it for standby.

AB-8 macroporous resin is placed in the chromatographic column, three kinds of polyphenol extracts are passed through the column to make polyphenols fully adsorbed on the resin, the resin column is washed with deionized water to remove the non-adsorbed impurities, eluted with 50% ethanol solution, the eluent is collected in a conical flask, concentrated by a rotary evaporator and lyophilized to obtain purified polyphenols. The freeze-dried polyphenol samples were weighed to 1 mg and dissolved in 50% ethanol. The content of polyphenols was determined according to the method in 2.5, and the purity of polyphenols was calculated using the following equation:

$$\mathrm{Purity}\;(\%)\;=\frac{M_{P}}{M_{t}}\times 100\%$$

where *M_P_* represents the mass of polyphenols in the tested sample (expressed as mg GAE) as determined by the standard curve, and *M_t_* represents the total dry mass of the weighed freeze-dried purified sample.

### 2.7. Identification of Polyphenol Components by HPLC-MS

The polyphenol components were determined and analyzed by referring to the previous research methods and slightly modified [21]. The 200 μL sample was filtered through the filter membrane and placed in the 200 μL intubation for detection. To ensure the reliability of the mass spectrometry data and to monitor potential system or environmental contamination, solvent blanks (50% ethanol) and procedural blanks were routinely injected between sample sequences. Background subtraction was applied during data processing to minimize the interference of solvent impurities and column carryover.

Chromatographic conditions: Thermo Scientific U3000 rapid liquid chromatography and reversed-phase chromatographic column were used for analysis. Chromatographic column: Waters HSS T3; mobile phase: positive mode was phase A (water 0.1% formic acid) and phase B (acetonitrile 0.1% formic acid), negative mode was phase A (water) and phase B (acetonitrile); elution gradient is shown in Table 1; the flow rate was 0.4 mL/min; the injection volume was 1 μL; column temperature 50 °C.

Mass spectrometry conditions: quadrupole electrostatic orbital trap mass spectrometer (Orbitrap Exploris 480) equipped with a thermoelectric spray ion source was used for mass spectrometry analysis; ion source settings: positive ion voltage 3500 V; negative ion voltage 2500 V; sheath gas flow 50; auxiliary air flow 10; purge gas flow 1; the temperature of ion transfer tube is 325 °C; vaporization chamber temperature 350 °C; full scan parameters: resolution 60,000; scanning range 67–1000 *m*/*z*; RF lens voltage 40%; automatic gain control target 100%; maximum injection time 100 ms; intensity threshold 5.0 × 10^3^; data-dependent secondary mass spectrometry parameters: isolation window 1 *m*/*z*; standardization of collision energy mode; HCD collision energy 20%, 40%, 60%; resolution 30,000; maximum injection time 54 ms; microscanning times 1.

### 2.8. Determination of Antioxidant Activity

Sample preparation: 1 mg, 2 mg, 3 mg, 4 mg of vitamin C standard and different polyphenol samples were accurately weighed. Vitamin C was dissolved in deionized water to 25 mL, and polyphenol samples were dissolved in 50% ethanol to 25 mL to obtain vitamin C solution and polyphenol sample solution with the concentration of 0.04 mg/mL, 0.08 mg/mL, 0.12 mg/mL, 0.16 mg/mL, respectively, for the determination of DPPH· scavenging rate, ABTS^+^· radical scavenging rate and total reducing power.

#### 2.8.1. DPPH· Scavenging Activity

We accurately weighed 15.8 mg of DPPH powder into a test tube, fully dissolved it with anhydrous ethanol, and then diluted the solution to 100 mL in a brown volumetric flask to obtain a 400 μmol/L DPPH working solution, which was stored protected from light. The UV spectrophotometer was started and preheated for 20 min. The blank tube, blank reference tube, sample tube, and sample reference tube were set up according to Table 2. The solutions were added in sequence, mixed evenly, and allowed to stand at room temperature in the dark for 30 min, after which the absorbance was measured at a wavelength of 517 nm, The vitamin C group was set as the positive control [22].

DPPH· scavenging rate is calculated according to the following Formula (1):

(1) $$\mathrm{DPPH}\cdot \;\mathrm{scavenging}\;\mathrm{rate}\;(\%)\;=\frac{\left(A_{1}-A_{2}\right)-\left(A_{3}{-A}_{4}\right)}{A_{1}-A_{2}}\;\times \;100\%$$

#### 2.8.2. ABTS^+^· Scavenging Activity

Different concentrations of polyphenol samples were prepared, and 0.1 mL was added to the test tube, and then 3.9 mL of ABTS^+^· solution was added to the test tube, which was recorded as *A*_1_. The blank group *A*_0_ used deionized water instead of polyphenol samples, while the control group *A*_2_ used PBS buffer instead of ABTS^+^· solution. Vitamin C was used as positive control. Then, the above solution was reacted at room temperature for 6 min, and the absorbance was measured at 734 nm [23].

The calculation method of ABTS^+^· scavenging rate is shown in Formula (2):

(2) $${\mathrm{ABTS}}^{+}\cdot \;\mathrm{scavenging}\;\mathrm{rate}\;(\%)\;=\left[1-\frac{\left(A_{1}-A_{2}\right)}{A_{0}}\right]\times 100\%$$

#### 2.8.3. Determination of Total Reducing Power

The prepared polyphenol samples of different concentrations were each drawn up to 1 mL and transferred into a test tube. Then, 2 mL of 0.2 mol/L phosphate buffer (pH 6.6) and 1% potassium ferricyanide solution were added, mixed evenly, and reacted at 50 °C for 20 min. After cooling to room temperature, 2 mL of 10% trichloroacetic acid was added, mixed evenly, and left to stand for 10 min. Accurately taking 2 mL of mixed solution, adding 2 mL of deionized water and 0.5 mL of 0.1% ferric chloride solution, after mixing evenly and standing for 10 min, the absorbance was measured at 700 nm. The higher the absorbance, the stronger the reducibility of polyphenol samples [24].

### 2.9. Determination of Anti Inflammatory Activity of Polyphenols

#### 2.9.1. Experimental Preparation

The following reagents and media were prepared before the experiment: DMEM complete medium containing 10% fetal bovine serum (FBS) and 1% penicillin streptomycin (P/S), and cell cryopreservation solution composed of 55% DMEM, 40% FBS and 5% dimethyl sulfoxide (DMSO).

A 20 mg polyphenol sample was accurately weighed, dissolved in DMEM containing 1% P/S (serum-free), filtered through 0.22 μm membrane, and then diluted with 1% DMEM to achieve the required concentration gradient. In addition, 1% DMEM was used to prepare the mother liquor of lipopolysaccharide (LPS), with the preparation concentration of 1 μg/mL. Finally, 10 μL trypan blue was mixed with 90 μL cell suspension for cell counting.

#### 2.9.2. Determination of Polyphenols on the Survival Rate of RAW264.7 Cells

To evaluate whether treatment with different concentrations of polyphenol samples affects the normal growth of RAW264.7 cells, a CCK-8 cytotoxicity assay was performed [25]. A cell suspension was prepared at a density of 2 × 10^5^ cells/mL, and 200 μL aliquots were seeded into 96-well plates. The plates were incubated at 37 °C in the 5% CO_2_ incubator for 24 h. After discarding the supernatant, the cells were divided into three groups: (a) negative control (NC) group, which received 100 μL of 1% medium; (b) LPS group, which was treated with 100 μL of LPS (1 μg/mL) to induce an inflammatory response; (c) drug groups, which were treated with 100 μL of polyphenol sample solutions at concentrations of 10, 20, 30, 40, and 50 μg/mL. Following 1 h of incubation, 100 μL of 1% medium was added to each well, and the cells were further incubated for 24 h. The supernatant was then discarded, and 10 μL of CCK-8 reagent was added to each well. After incubation at 37 °C for 30 min, the absorbance was measured at 450 nm using a microplate reader to assess cell viability.

(3) $$\mathrm{Cell}\;\mathrm{viability}\;(\%)=\frac{A_{sample}-A_{blank}}{A_{control}-A_{blank}}\times 100$$

where *A_sample_* is the absorbance of the experimental well. *A_control_* is the absorbance of the untreated control well. *A_blank_* is the absorbance of the blank well.

#### 2.9.3. Determination of NO Production in RAW264.7 Cells

To assess nitric oxide (NO) production, RAW264.7 cells were seeded into 96-well plates at a density of 3 × 10^5^ cells/mL (200 μL per well) and incubated at 37 °C in a 5% CO_2_ atmosphere for 24 h. Following the removal of the supernatant, the cells were divided into three groups: (a) the normal control (NC) group, which received standard medium; (b) the LPS model group, which was treated with LPS to induce an inflammatory response; and (c) the sample groups, which were pre-treated with 100 μL of the polyphenol extracts at varying concentrations (10, 20, 30, 40, and 50 μg/mL). After 1 h of pre-incubation, LPS (final concentration of 1 μg/mL) was added to both the LPS and sample groups, followed by an additional 24 h of incubation.

Subsequently, 100 μL of the culture supernatant was collected from each well and mixed with an equal volume of Griess reagent. After a 10 min reaction at room temperature in the dark, the absorbance was measured at 540 nm using a microplate reader. To accurately quantify the NO levels, a standard curve was established concurrently using serially diluted sodium nitrite ($NaNO_{2}$) standard solutions. The calibration curve exhibited excellent linearity over the concentration range of 0 to 100 μmol/L ($R^{2}>0.996$). The absolute NO concentration in the cell supernatant was calculated based on the linear regression equation derived from this standard curve and expressed in μM. Finally, the relative NO production rate was calculated according to Formula (4):

(4) $$NO\;\mathrm{production}\;\left(\%\right)\;=\;\frac{C_{drug}}{C_{LPS}}\times 100$$

where *C_drug_* and *C_LPS_* represent the NO concentrations in the sample-treated group and the LPS-stimulated group, respectively.

### 2.10. Determination of Inhibitory Activity of Polyphenols on α-Glucosidase

The α-glucosidase inhibitory activity was evaluated following standard enzymatic assay protocols with complete methodological parameters [26]. The α-glucosidase enzyme solution (14.00 nKat/mL) and the substrate PNPG (5.0 mM) were both prepared in 0.1 M phosphate buffer (pH 6.8).

Briefly, 5.0 mL of phosphate buffer (pH 6.8), 1.0 mL of the α-glucosidase solution (14.00 nKat/mL), and 0.5 mL of the polyphenol sample solutions at varying concentrations (1–40 mg/mL) were accurately pipetted into test tubes. The mixtures were pre-incubated at 37 °C for 10 min to allow sufficient enzyme-inhibitor binding. The catalytic reaction was subsequently initiated by the addition of 1.0 mL of the PNPG solution and maintained at 37 °C for exactly 10 min. Finally, the reaction was terminated by adding 4.0 mL of 0.2 mol/L $Na_{2}CO_{3}$ solution. The absorbance of the reaction mixtures was measured at 405 nm, with acarbose serving as the positive control group. Equation (5) shows the calculation formula of *α*-glucosidase inhibitory activity:

(5) $$Inhibitory\;activity\left(\%\right)=\left[1-\frac{A_{sample}-A_{sample\_blank}}{A_{control}-A_{blank}}\right]\times 100$$

where $A_{sample}$ is the absorbance of the reaction mixture containing the polyphenol sample, enzyme, and substrate. $A_{sample\_blank}$ is the absorbance of the sample background, containing the polyphenol sample and substrate, but no enzyme. $A_{control}$ is the absorbance of the control reaction containing the enzyme and substrate, but no polyphenol sample. $A_{blank}$ is the absorbance of the system background containing only the substrate and buffer, without enzyme or sample.

### 2.11. Determination of Antibacterial Activity of Polyphenols

#### 2.11.1. Strain Cultivation

*Staphylococcus aureus*, *Escherichia coli*, and *Bacillus subtilis* were cultured. The LB solid medium was prepared by dissolving tryptone (10.0 g), sodium chloride (10.0 g), yeast extract (5.0 g), and agar powder (20.0 g) in deionized water (1 L). The pH of the medium was adjusted to 7.4, following which it was sterilized by autoclaving at 121 °C for 20 min. The LB liquid medium was prepared using the same formulation, except that the agar powder was omitted. All cultures were incubated at 37 °C. The bacterial strains used in this study were all sourced from the China Industrial Culture Collection (CICC).

#### 2.11.2. Determination of Antibacterial Halo Size

The agar diffusion method was used to determine the diameter of the antibacterial zone of plant polyphenols. A bacterial suspension (100 μL) was spread onto the corresponding solid medium (90 mm in diameter). Wells (6 mm in diameter) were punched into the agar using a sterile stainless steel punch, and the agar plugs were removed with an inoculating needle. The wells were then filled with plant polyphenol solutions at various concentrations (100 μL each). The Petri dishes were placed upright in an incubator and incubated for 24 h. The antibacterial activity was expressed as the diameter (in mm) of the inhibition zone produced around each well. All of the above procedures were performed under sterile conditions.

#### 2.11.3. Determination of Minimum Inhibitory Concentration

The minimum inhibitory concentration (MIC) was determined using the two-fold broth microdilution method. In the first column of a 96-well plate, 100 μL of the peach residue polyphenol sample solution was added. Subsequently, 100 μL of the mixture was transferred to the second column, and 100 μL of liquid medium was added to mix and dilute the sample serially into the subsequent columns. Finally, 100 μL of bacterial suspension at a concentration of 10^6^ CFU/mL was added to each well. The positive controls were also included, tetracycline hydrochloride (THC) was used as a reference control for the antibacterial assay. The plate was incubated at 37 °C for 24 h, after which the turbidity of the culture medium in each well was examined. The lowest concentration of the peach residue polyphenol sample that resulted in no visible turbidity was recorded as the MIC.

### 2.12. Statistical Analysis

All extraction processes were performed using three independent experimental batches (biological replicates), and all subsequent analytical measurements and in vitro assays were conducted in technical triplicates. The data were expressed as mean ± standard deviation (SD). Statistical analysis was conducted using SPSS 26.0 software. One-way analysis of variance (ANOVA) followed by Duncan’s multiple range test was employed to assess differences among groups, with statistical significance (set at p<0.05) determined based on Duncan’s multiple range test. Graphs were generated using Origin 2019b software.

## 3. Results and Discussion

### 3.1. Effects of Different Grinding Methods on the Content and Purity of Polyphenols

**Figure 1:**
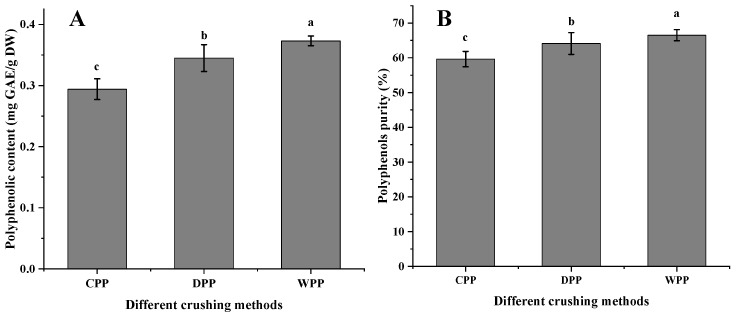
The effect of different crushing methods on polyphenol content (**A**) and purity (**B**). Note: Significant differences are indicated by lowercase letters, as determined by Duncan’s multiple range test.

Polyphenols are sensitive to environmental conditions and processing methods, and different treatment methods will affect their dissolution rate and stability [27]. It can be seen from Figure 1A,B that there are significant differences in the content and purity of polyphenols in peach pomace treated by different ultrafine grinding methods [28]. Compared with CPP, ultrafine grinding significantly increased the dissolution of polyphenols, mainly because ultrafine grinding technology induced severe mechanical damage to plant cell walls and profound changes to the food matrix, thus promoting the rapid dissolution and release of embedded intracellular phytochemicals [29,30]. After purification by macroporous resin, the purity of polyphenols obtained by WPP was the highest, which was significantly better than that of DPP and CPP. This was mainly due to the fact that the liquid environment in the process of wet ultrafine grinding can dissipate the local heat generated by mechanical grinding in time, thus reducing the degradation of heat-sensitive polyphenols. At the same time, the infiltration of liquid phase is helpful to maintain the stability of polyphenols in the extraction system and avoid oxidative polymerization [31]. In addition, the high-strength wet pulverization makes the cells more thoroughly broken and the contents of cells more fully dissolved, thus obtaining higher purity phenolic substances in the subsequent purification process.

Furthermore, the efficiency and mass balance of the AB-8 resin purification step for the WPP sample were quantitatively evaluated. The purification yield, defined as the mass ratio of the final lyophilized purified extract to the initial crude extract loaded onto the column, was determined to be approximately 24.5%. This significant mass reduction is consistent with the mass balance profile, which indicates that a large proportion of highly polar, non-target impurities (such as soluble sugars, organic acids, and soluble proteins) were effectively washed out during the deionized water rinsing phase. Meanwhile, the recovery rate of polyphenols, calculated as the ratio of the total polyphenol mass in the purified desorbed fraction to the total polyphenol mass in the loaded crude extract, reached 81.2%. These solid mass balance data demonstrate that the AB-8 resin protocol utilized in this study not only drastically enhanced the polyphenol purity but also maintained a high recovery efficiency, validating its potential for large-scale upcycling applications.

### 3.2. HPLC-MS Analysis

**Figure 2:**
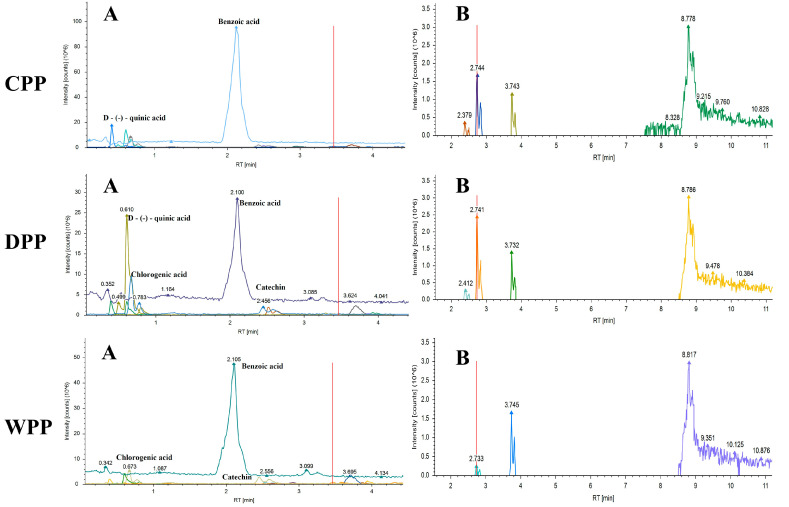
HPLC-MS chromatogram of polyphenol extract under different grinding methods: (**A**) positive ion mode; (**B**) negative ion mode.

In order to further clarify the specific composition of polyphenols, HPLC-MS analysis was carried out. The chromatogram of polyphenol extract of the sample is shown in Figure 2, and the identification analysis of components is shown in Table 3. The HPLC-MS chromatogram of the three polyphenol extract samples is shown in Figure 2. It can be seen from the figure that 15 main polyphenols were detected in the polyphenol extract of peach pomace, among which benzoic acid, D-(-)-quinic acid, chlorogenic acid, catechin, dibutyl phthalate and other substances with high content were detected.

Table 3 provides the detailed identification analysis of the 15 peaks detected in the polyphenol samples. The results showed that HPLC-MS analysis identified a total of 15 main components, including typical polyphenolic substances such as chlorogenic acid, quinic acid, catechins, and quercetin derivatives, as well as some organic acids (such as benzoic acid and salicylic acid) and other compounds (such as dibutyl phthalate). It is important to clarify that dibutyl phthalate is a widespread industrial plasticizer rather than a natural plant metabolite. Its detection in the extract is attributed to exogenous contamination, likely introduced from plastic lab consumables, tubing, or resin treatments during the extraction and purification processes, and it does not contribute to the inherent bioactivity of the peach pomace.

By comparing the peak areas of the same individual compound across different pretreatment groups, the relative extraction efficiencies of the three methods could be evaluated. For instance, the peak areas of chlorogenic acid, D-(-)-quinic acid, and catechin followed the order of DPP > CPP > WPP. This indicates that the dry superfine grinding treatment significantly improved the extraction efficiency for these specific components compared to the wet or coarse grinding methods. Conversely, for compounds such as quercetin-3-*β*-D-glucoside and isorhamnetin-3-O-rutinoside, the peak areas exhibited an order of WPP > DPP > CPP, suggesting that wet ultrafine grinding was more conducive to the release or preservation of these specific flavonoid glycosides, possibly due to the aqueous medium promoting the dissolution of polar components and buffering the mechanical heat. Furthermore, rutin, 2-chlorobenzoic acid, and 3-methylsalicylic acid were either not detected or showed extremely low peak areas under dry ultrafine pulverization. This is likely because the localized high temperatures generated by mechanical friction during dry grinding led to the degradation of these heat-sensitive components. In contrast, the relatively mild environment and liquid medium provided by wet ultrafine pulverization were highly beneficial for retaining these compounds.

It is noteworthy that while the peak areas of certain low-molecular-weight monomers (such as chlorogenic acid and catechin) were highest in the DPP extract, the overall total polyphenol content (TPC) determined by the Folin–Ciocalteu assay was favored by the WPP treatment. This apparent discrepancy stems from the fundamental differences in the analytical targets of the two methods. The HPLC-MS specifically profiles identifiable small-molecule phytochemicals, whereas the TPC assay captures the bulk reducing capacity of all phenolic hydroxyl groups. The intense dry mechanical stress in DPP efficiently released smaller phenolics; however, the WPP treatment—aided by its low-temperature aqueous environment—not only prevented the thermal degradation of heat-sensitive highly polar flavonoid glycosides (e.g., rutin and quercetin derivatives) but also likely facilitated the substantial co-extraction of water-soluble polymeric phenolics. These complex polyphenols, although not individually resolved in the targeted HPLC-MS profile, contributed significantly to the superior overall TPC observed in the WPP extract.

In conclusion, dry ultrafine grinding is more suitable for extracting basic polyphenols (such as chlorogenic acid, catechin, etc.). The extraction efficiency of some flavonoid glycosides (such as rutin, quercetin derivatives, etc.) was higher by wet ultrafine grinding. The overall extraction efficiency of coarse grinding is in the middle, but the operation is simple, which is suitable for scenes that do not require high extraction efficiency.

### 3.3. Effect of Different Grinding Methods on Antioxidant Activity of Polyphenols

**Figure 3:**
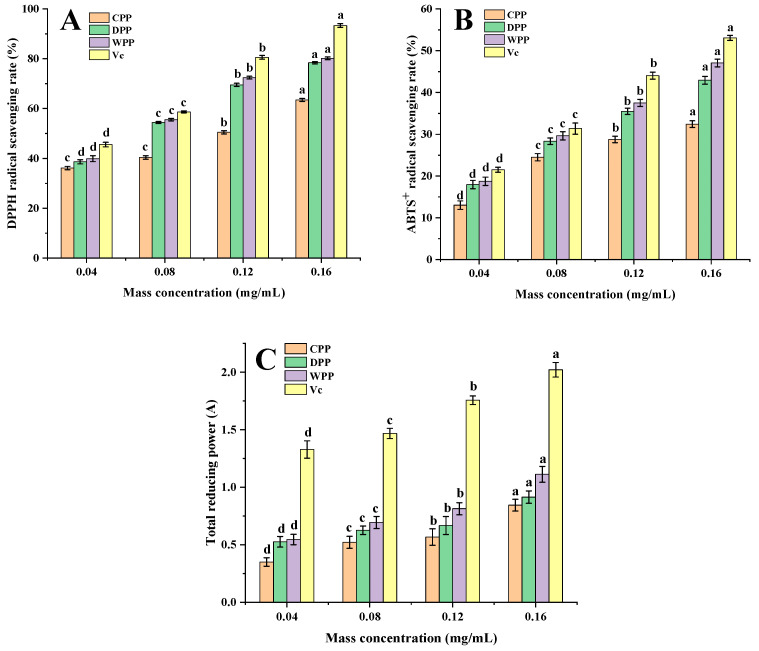
Effect of different grinding methods on antioxidant activity of polyphenols: (**A**) DPPH· radical scavenging rate; (**B**) ABTS^+^· radical scavenging rate; (**C**) total reducing power. Note: Significant differences are indicated by lowercase letters, as determined by Duncan’s multiple range test.

Currently, DPPH·, ABTS^+^· and total reducing power are three free radical models commonly used to measure the antioxidant potential of polyphenols [32]. Based on this, we can fully clarify the differences in antioxidant activity of polyphenols prepared by different grinding methods, and provide reference for selecting appropriate grinding methods. As shown in Figure 3, in the three antioxidant evaluation systems, the polyphenol samples extracted by different crushing treatments have obvious antioxidant activity, and the antioxidant capacity has a great relationship with the mass concentration of the sample and the crushing method. DPPH· scavenging rate, ABTS^+^· scavenging rate and total reducing capacity of polyphenols were positively correlated with polyphenol concentration. The higher the polyphenol concentration, the higher the scavenging rate and total reducing capacity, and the stronger the antioxidant capacity. With the same mass concentration of polyphenols, the DPPH· scavenging rate, ABTS^+^· scavenging rate and total reducing power of WPP were slightly higher than DPP, and the lowest was CPP. The DPPH·and ABTS^+^· scavenging rate of WPP were slightly lower than that of vitamin C. Therefore, the crushing treatment had a certain effect on the antioxidant activity of polyphenols in peach pomace, and WPP had the best antioxidant effect, followed by DPP, both of which had a better antioxidant effect.

This is mainly due to the collaborative optimization of the extraction efficiency and structural integrity of polyphenols by its process characteristics: the strong shear in the liquid medium realizes the deep wall breaking at the cell level, which significantly improves the mass transfer efficiency and dissolution rate of polyphenols [33]. At the same time, the thermal buffering and isolation of the medium inhibited the destruction of polyphenol hydroxyl structure by mechanical friction heat and oxidation environment, and stabilized its conformation through hydrogen bonding, thus retaining the biological activity of polyphenols to the greatest extent. In addition, the superfine treatment increased the specific surface area and promoted the electron transfer and free radical contact in the antioxidant reaction [34,35]. Therefore, WPP has higher polyphenol purity and stronger reduction ability, which makes its DPPH· scavenging rate and ABTS^+^· scavenging rate at the same mass concentration significantly better than that of coarse grinding and dry ultrafine grinding samples.

### 3.4. Effect of Different Grinding Methods on Anti Inflammatory Activity of Polyphenols

#### 3.4.1. Effect of Polyphenols from Peach Pomace on the Survival Rate of Raw 264.7 Cells

**Figure 4:**
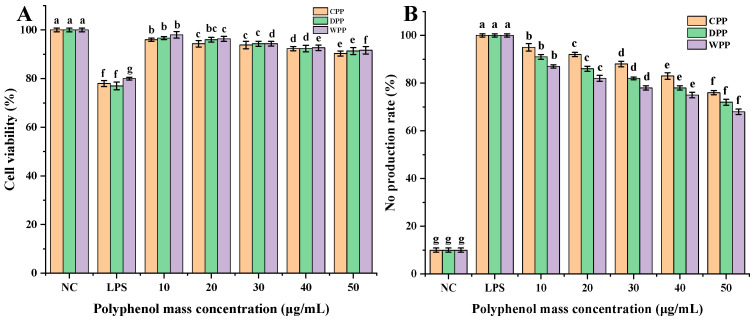
Effects of polyphenols with different concentrations and grinding methods on the survival rate (**A**) and NO production (**B**) of raw 264.7 cells. Note: Significant differences are indicated by lowercase letters, as determined by Duncan’s multiple range test.

It can be seen from Figure 4A that within the concentration range of 10–50 μg/mL polyphenols, the cell survival rate was more than 90%, and no obvious cytotoxicity was observed. At different concentrations, the cell survival rate of WPP was slightly higher than that of DPP, with less cytotoxicity and higher cell survival rate. Therefore, the concentration of polyphenols in peach pomace treated by different crushing methods in subsequent experiments can be selected as 50 μg/mL or less for cell experiments.

#### 3.4.2. Effect of Polyphenols from Peach Pomace on NO Production in Raw 264.7 Cells

As shown in Figure 4B, nitric oxide (NO) production in the LPS-stimulated group was significantly higher than that in the normal control (NC) group, confirming the successful establishment of the LPS-induced RAW264.7 cell inflammation model. Following treatment with the extracts, NO production decreased in a dose-dependent manner across all groups, indicating that the extracts possessed notable in vitro anti-inflammatory activity [36,37]. At equivalent concentrations, the inhibitory effect on NO production followed the order of WPP > DPP > CPP. This demonstrates that the extract prepared by wet ultrafine grinding exhibited the most potent anti-inflammatory efficacy. The superior performance of the WPP extract is consistent with the HPLC-MS results (Section 3.2). The low-temperature liquid environment provided by the wet grinding process effectively buffered mechanical heat, thereby preventing the thermal degradation of highly bioactive and heat-sensitive flavonoids (such as rutin and quercetin derivatives). The improved preservation of these intact phytochemicals in the WPP extract likely contributed to its higher efficacy in attenuating NO release while maintaining high cell viability in the RAW264.7 model.

### 3.5. Effect of Different Grinding Methods on the Inhibitory Activity of Polyphenols on α-Glucosidase

**Figure 5:**
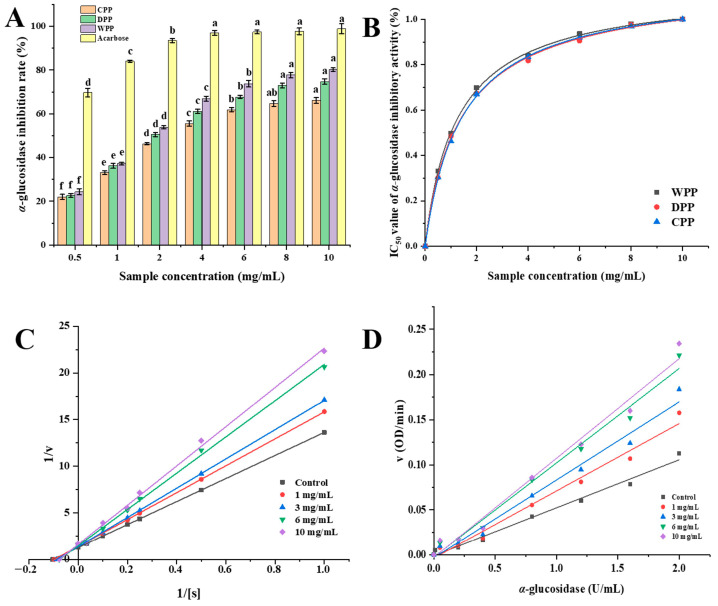
Analysis of *α*-glucosidase inhibitory activity of polyphenols from peach pomace ((**A**) inhibitory activity of different polyphenolic samples on *α*-glucosidase, (**B**) IC_50_ of *α*-glucosidase inhibitory activity, (**C**) Lineweaver–Burk diagram of WPP polyphenol on *α*-glucosidase, (**D**) reversibility analysis of WPP polyphenol and *α*-glucosidase). Note: Significant differences are indicated by lowercase letters, as determined by Duncan’s multiple range test.

Based on the research of Dai et al. [38] it was determined that polyphenol extract from peach by-product had significant hypoglycemic properties. The inhibitory activity of polyphenols from peach pomace on *α*-glucosidase is a key index to measure its therapeutic potential for diabetes [39]. Figure 5A shows the inhibitory activity of polyphenol samples with different concentrations on *α*-glucosidase. Within the concentration range of 0.5~10 mg/mL, the inhibitory activity of polyphenol samples prepared by different grinding methods showed a positive correlation with the dose. In the concentration range of 0.5~4 mg/mL, the inhibitory activity of polyphenols on *α*-glucosidase increased significantly with the increase in concentration. In the concentration range of 4–10 mg/mL, the enzyme inhibitory activity of polyphenol samples increased slowly. Figure 5B shows the IC50 values of three polyphenol samples for *α*-glucosidase inhibitory activity, which were WPP: 1.30, DPP: 1.47 and CPP: 1.49 respectively. It can be seen that under the condition of the same substrate concentration, the crushing method affects the inhibitory activity of polyphenols to a certain extent. By comparison, wet ultrafine grinding had the best inhibitory effect on *α*-glucosidase.

In addition, the relationship between the concentration of WPP on *α*-glucosidase and the reaction rate was evaluated, and the types and mechanisms of WPP inhibition on *α*-glucosidase were explored. It can be seen from Figure 5C that the inhibition curves of WPP solutions with different concentrations intersect the Y axis, and with the increase in WPP concentration, the intercept of the double reciprocal curve intersecting the X axis gradually decreases. In addition, when the maximum reaction rate was constant, the Michaelis coefficient gradually increased with the increase in WPP concentration, which indicated that WPP was a competitive inhibition of *α*-glucosidase [40]. Figure 5D evaluated the reversibility of the WPP sample on α-glucosidase inhibition. The plots of the initial reaction rate versus enzyme concentration at different WPP concentrations yielded a family of straight lines that all passed through the origin (0, 0). The gradient (slope) of these lines decreased with increasing WPP sample concentration. According to standard enzyme kinetics, this specific graphical pattern confirms that the inhibitor reduces the apparent enzyme activity without permanently titrating or covalently modifying the active sites, indicating that the inhibition of WPP on α-glucosidase was reversible [41]. In conclusion, combining the results from the Lineweaver–Burk plot and the reversibility assay, WPP acts as a reversible competitive inhibitor of α-glucosidase.

### 3.6. Effect of Different Crushing Methods on the Antibacterial Activity of Polyphenols from Peach Pomace

It can be seen from Table 4 and Table 5 that the polyphenols from peach pomace prepared by different crushing methods have a certain degree of inhibition on *Staphylococcus aureus*, *Escherichia coli* and *Bacillus subtilis*, and different crushing methods have a significant impact on the diameter of their inhibition zone. As shown in Table 4, WPP had the strongest inhibitory activity against *Staphylococcus aureus*, *Escherichia coli* and *Bacillus subtilis*, with inhibition zone diameters of 10.83, 11.32 and 4.33 respectively, followed by DPP, with inhibition zone diameters of 10.15, 10.77 and 4.02 respectively, and CPP had the lowest inhibitory activity. In addition, it can be seen from Table 5 that different crushing methods have an impact on the minimum inhibitory concentrations (MIC) of three bacteria. The MIC of WPP and DPP to *Staphylococcus aureus*, *Escherichia coli* and *Bacillus subtilis* were 3.13 3.13 12.50, indicating that WPP and DPP were more conducive to inhibiting the proliferation of *Staphylococcus aureus* and *Escherichia coli*. In addition, the antibacterial spectrum of peach pomace polyphenols against *Staphylococcus aureus*, *Escherichia coli* and *Bacillus subtilis* confirmed the strong and broad-spectrum defense ability of the stabilized peach pomace extract against major food-borne pathogens, which was consistent with the conclusion of Mihailova et al. [42].

In conclusion, both WPP and DPP exhibited identical MIC values (3.13, 3.13, and 12.50 mg/mL, respectively) against the tested strains, indicating that both ultrafine grinding methods yield extracts with highly potent and comparable intrinsic antibacterial efficacy. While WPP showed slightly larger inhibition zone diameters in the agar diffusion assay compared to DPP (e.g., 10.83 mm vs. 10.15 mm for *S. aureus*), this sub-millimeter difference, although statistically significant, is not considered biologically meaningful in terms of actual bactericidal power. Instead, this slight variation in the agar assay is more likely attributed to diffusion kinetics. As demonstrated in the HPLC-MS results (Section 3.2), the liquid-phase environment of wet pulverization effectively preserved highly polar, water-soluble flavonoid glycosides. This increased hydrophilicity likely enhanced the diffusion rate of the WPP extract through the aqueous agar matrix, resulting in a visually larger inhibition zone. Ultimately, both dry and wet ultrafine grinding significantly outperformed the coarse grinding treatment (CPP), proving to be highly effective strategies for maximizing the antibacterial potential of peach pomace polyphenols.

## 4. Conclusions

The comprehensive value of fruit processing by-products is a key task in sustainable food science. The by-products of peach processing, especially the peel, as a solid substrate rich in a variety of free and bound polyphenols, have attracted more and more attention. However, how to efficiently release polyphenol bioactive substances from this dense plant matrix is still a technical bottleneck restricting its deep utilization. In this study, polyphenols were extracted from peach pomace by different ultrafine grinding methods, and their effects on polyphenol content, chemical composition, antioxidant and potential anti-inflammatory activities were systematically explored. The results showed that the dry ultrafine grinding was more suitable for extracting basic polyphenols, and the wet ultrafine grinding was more efficient for extracting some flavonoid glycosides. The content and purity of polyphenols in peach pomace obtained by wet ultrafine pulverization were higher, and the evaluation results of antioxidant and anti-inflammatory activities were better than those of dry processing, showing stronger functional potential. In addition, the inhibitory mechanism of polyphenols from peach pomace on *α*-glucosidase was also discussed. The results showed that wet superfine grinding was an effective method to improve the inhibitory activity of polyphenols on *α*-glucosidase, and its inhibition on *α*-glucosidase belonged to a reversible competitive inhibition mechanism. Finally, the antibacterial activities of polyphenols from peach pomace prepared by three grinding methods against *Staphylococcus aureus*, *Escherichia coli* and *Bacillus subtilis* were analyzed, and WPP had the best antibacterial activity. Although dry ultrafine grinding also has good antioxidant and anti-inflammatory effects, wet ultrafine grinding has significant advantages in improving the extraction efficiency and biological effects of polyphenol active ingredients, and is a better choice to realize the high-value utilization of peach pomace. This study provides a theoretical basis and scientific basis for the efficient extraction and deep development of polyphenols from peach pomace.

While wet ultrafine grinding significantly improves extraction yields and biological activities, it is important to acknowledge the potential limitations regarding its practical application. The high moisture content associated with wet-processed materials may compromise storage stability and shelf life, potentially increasing the risk of microbial growth if the materials are not promptly dehydrated (e.g., via freeze-drying) or utilized. Although evaluating the storage stability of the wet-pulverized material is beyond the scope of the current work, future studies should focus on assessing microbial safety and optimizing preservation methods to ensure the long-term stability of these materials for industrial application.

## Figures and Tables

**Table 1:** Reversed phase chromatographic elution procedure.

Time (min)	A (v%)	B (v%)
0	95	5
0.5	95	5
5.5	50	50
9	5	95
10.5	5	95
10.6	95	5
12	95	5

**Table 2:** DPPH method sample addition details.

Reagent	*A*_1_ (Blank)	*A*_2_ (Blank Reference)	*A*_3_ (Sample)	*A*_4_ (Sample Reference)
Sample	0	0	0.2 mL	0.2 mL
Ethanol	1 mL	2 mL	0.8 mL	1.8 mL
Deionized water	2 mL	2 mL	2 mL	2 mL
DPPH	1 mL	0	1 mL	0

**Table 3:** Identification results of the main compounds detected in the extract.

/	RT (min)	MS (*m*/*z*)	CPP (PA)	DPP (PA)	WPP (PA)	Component
1	0.621	353.08819	65,469,440	101,825,474	15,897,446	Chlorogenic acid
2	0.611	191.05638	5,143,107	13,120,547	2,200,609	D-(-)-quinic acid
3	2.518	289.0722	5,398,910	6,443,717	2,784,534	Catechin
4	0.504	353.08821	7,444,518	10,906,652	1,870,822	Neochlorogenic acid
5	2.117	121.02962	1,090,883,351	401,193,425	632,888,474	Benzoic acid
6	0.671	137.02461	33,056,734	31,681,422	19,236,507	Salicylic acid
7	3.706	463.08897	16,859,460	17,958,031	30,042,533	Quercetin-3beta-D-glucoside
8	2.938	154.99078	8,107,490	-	5,526,997	2-Chlorobenzoic acid
9	3.582	609.14697	361,405	-	5,048,103	Rutin
10	3.467	151.04031	5,815,383	-	4,710,573	3-Methylsalicylic acid
11	0.815	153.01958	6,100,678	10,406,745	4,582,089	2,3-Dihydroxybenzoic acid
12	3.936	623.16274	3,436,849	3,776,479	6,697,702	Isorhamnetin-3-O-rutinoside
13	3.865	593.15218	-	1,702,018	3,399,094	Kaempferol-3-O-rutinoside
14	3.738	545.19961	3,250,638	3,996,285	4,800,153	Lariciresinol 4-O-glucoside
15	8.794	279.15911	89,816,357	79,527,537	76,328,431	Dibutyl phthalate

PA represents the integrated peak area of the corresponding compounds detected by HPLC-MS. Identification criteria: The phenolic compounds were tentatively identified by comparing their retention times (RT) and accurate precursor mass-to-charge ratios (m/z) with the relevant literature and mass spectral databases. “-” indicates that the compound was not detected in the sample.

**Table 4:** Inhibition zone diameter of polyphenol extract from peach pomace prepared by different grinding methods on tested bacteria.

Extracts	Diameter of the Inhibition Zone/mm		
	*Staphylococcus aureus*	*Escherichia coli*	*Bacillus subtilis*
CPP	9.66 ± 0.23 ^d^	10.12 ± 0.14 ^c^	3.78 ± 0.11 ^c^
DPP	10.15 ± 0.51 ^c^	10.77 ± 0.25 ^bc^	4.02 ± 0.16 ^bc^
WPP	10.83 ± 0.27 ^b^	11.32 ± 0.18 ^b^	4.33 ± 0.37 ^b^
THC	14.21 ± 0.21 ^a^	16.21 ± 0.09 ^a^	8.67 ± 0.15 ^a^

Note: Significant differences are indicated by lowercase letters, as determined by Duncan’s multiple range test.

**Table 5:** Effects of polyphenols from peach pomace prepared by different grinding methods on three kinds of polyphenols minimum inhibitory concentration of bacteria.

Extracts	MIC (mg/mL)		
	*Staphylococcus aureus*	*Escherichia coli*	*Bacillus subtilis*
CPP	6.25	6.25	25
DPP	3.13	3.13	12.50
WPP	3.13	3.13	12.50
THC	<0.75	<0.75	1.50

## Data Availability

The original contributions presented in the study are included in the article; further inquiries can be directed to the corresponding author.
